# Leveraging the genetic correlation between traits improves the detection of epistasis in genome-wide association studies

**DOI:** 10.1093/g3journal/jkad118

**Published:** 2023-05-27

**Authors:** Julian Stamp, Alan DenAdel, Daniel Weinreich, Lorin Crawford

**Affiliations:** Center for Computational Molecular Biology, Brown University, Providence, RI 02906, USA; Center for Computational Molecular Biology, Brown University, Providence, RI 02906, USA; Center for Computational Molecular Biology, Brown University, Providence, RI 02906, USA; Department of Ecology, Evolution, and Organismal Biology, Brown University, Providence, RI 02906, USA; Center for Computational Molecular Biology, Brown University, Providence, RI 02906, USA; Department of Biostatistics, Brown University, Providence, RI 02903, USA; Microsoft Research New England, Cambridge, MA 02142, USA

**Keywords:** epistasis, complex traits, covariance component analysis, multivariate linear mixed models

## Abstract

Epistasis, commonly defined as the interaction between genetic loci, is known to play an important role in the phenotypic variation of complex traits. As a result, many statistical methods have been developed to identify genetic variants that are involved in epistasis, and nearly all of these approaches carry out this task by focusing on analyzing one trait at a time. Previous studies have shown that jointly modeling multiple phenotypes can often dramatically increase statistical power for association mapping. In this study, we present the “multivariate MArginal ePIstasis Test” (mvMAPIT)—a multioutcome generalization of a recently proposed epistatic detection method which seeks to detect *marginal epistasis* or the combined pairwise interaction effects between a given variant and all other variants. By searching for marginal epistatic effects, one can identify genetic variants that are involved in epistasis without the need to identify the exact partners with which the variants interact—thus, potentially alleviating much of the statistical and computational burden associated with conventional explicit search-based methods. Our proposed mvMAPIT builds upon this strategy by taking advantage of correlation structure between traits to improve the identification of variants involved in epistasis. We formulate mvMAPIT as a multivariate linear mixed model and develop a multitrait variance component estimation algorithm for efficient parameter inference and *P*-value computation. Together with reasonable model approximations, our proposed approach is scalable to moderately sized genome-wide association studies. With simulations, we illustrate the benefits of mvMAPIT over univariate (or single-trait) epistatic mapping strategies. We also apply mvMAPIT framework to protein sequence data from two broadly neutralizing anti-influenza antibodies and approximately 2,000 heterogeneous stock of mice from the Wellcome Trust Centre for Human Genetics. The mvMAPIT R package can be downloaded at https://github.com/lcrawlab/mvMAPIT.

## Introduction

Genome-wide association (GWA) studies have contributed substantially in the discovery of genetic markers associated with the architecture of disease phenotypes (Ripke *et al.* 2014; Ellinghaus *et al.* 2016; Fuchsberger *et al.* 2016; Visscher *et al.* 2012, 2017; Loos 2020). Epistasis, commonly defined as the interaction between genetic loci, has long been thought to play a key role in defining the genetic architecture underlying many complex traits and common diseases (Tao *et al.* 2012; Wei *et al.* 2012; Kirino *et al.* 2013; Chen *et al.* 2017; Gusareva *et al.* 2018). Indeed, previous studies have detected pervasive epistasis in many model organisms (Peripato *et al.* 2004; Tong *et al.* 2004; Brem *et al.* 2005; Deutschbauer and Davis 2005; Kroymann and Mitchell-Olds 2005; Collins *et al.* 2006; Lehner *et al.* 2006; Onge *et al.* 2007; Wentzell *et al.* 2007; Shao *et al.* 2008; Flint and Mackay 2009; Gerke *et al.* 2009; Costanzo *et al.* 2010; He *et al.* 2010; Horn *et al.* 2011; Jarvis and Cheverud 2011; Leamy *et al.* 2011; Pettersson *et al.* 2011; Szappanos *et al.* 2011; Gaertner *et al.* 2012; Bloom *et al.* 2013; Chari and Dworkin 2013; Monnahan and Kelly 2015; Darnell *et al.* 2022). Substantial contributions of epistasis to phenotypic variance have been revealed for many complex traits (Huang and Mackay 2016; Crawford *et al.* 2018) and have been suggested to constitute an important component of evolution (Weinreich *et al.* 2006). Furthermore, modeling epistasis in addition to additive and dominant effects has been argued to increase phenotypic prediction accuracy in model organisms (Forsberg *et al.* 2017; Zhou *et al.* 2022) and facilitate genomic selection in breeding programs (Muñoz *et al.* 2014; Jiang and Reif 2015; Runcie *et al.* 2021). Despite a longstanding and currently ongoing debate about the contribution of nonadditive effects on the architecture of human complex traits (Long and Langley 1999; Marchini *et al.* 2005; Hill *et al.* 2008; Shao *et al.* 2008; Crow 2010; Zuk *et al.* 2012; Hivert *et al.* 2021; Pazokitoroudi *et al.* 2021; Wainschtein *et al.* 2022), recent genetic mapping studies have also identified evidence of epistatic interactions that significantly contribute to quantitative traits and diseases (Brown *et al.* 2014; Fang *et al.* 2019; van de Haar *et al.* 2019; Sheppard *et al.* 2021), and some have recently argued that gene-by-gene interactions can drive heterogeneity of causal variant effect sizes across diverse human populations (Patel *et al.* 2022). Importantly, epistasis is often proposed as a key contributor to missing heritability—the proportion of heritability not explained by the top associated variants in GWA studies (Wanstrat and Wakeland 2001; Eichler *et al.* 2010; Gusareva *et al.* 2014; Gusareva and Van Steen 2014; Gusareva *et al.* 2018).

Many statistical methods have been developed to facilitate the identification of epistasis in complex traits and diseases. Generally, these existing tools can be classified into two frameworks. In the first framework, explicit searches are performed to detect significant pairwise or higher order interactions. More specifically, they use various strategies including exhaustive search (Purcell *et al.* 2007; Schüpbach *et al.* 2010; Ma *et al.* 2013), probabilistic search (Prabhu and Pe’er 2012), or prioritization based on a predefined set of biological annotations of signaling pathways or genomic regulatory units (Lewinger *et al.* 2013; Guo *et al.* 2021). Different statistical paradigms have been implemented for these explicit search-based approaches including various frequentist tests (Purcell *et al.* 2007; Wan *et al.* 2010; Lishout *et al.* 2013), Bayesian inference (Zhang and Liu 2007; Tang *et al.* 2009; Zhang *et al.* 2011; Guo *et al.* 2019), and, most recently, detecting epistasis using machine learning (Chang *et al.* 2020; Fergus *et al.* 2020). Indeed, the explosion of large-scale genomic data sets has provided the unique opportunity to integrate many of these techniques as standard statistical tools within GWA analyses. Many modern GWA applications have data sets that can include hundreds of thousands of individuals genotyped at millions of markers and phenotyped for thousands of traits (Nagai *et al.* 2017; Bycroft *et al.* 2018). Due to the potentially large space of genetic interactions (e.g. J(J−1)/2 possible pairwise combinations for *J* variants in a study), explicit search-based methods often suffer from heavy computational burden. Even with various efficient computational improvements (Wan *et al.* 2010; Hemani *et al.* 2011; Prabhu and Pe’er 2012; Zhu and Fang 2018; Bayat *et al.* 2021), exploring over a large combinatorial domain remains a daunting task for many epistatic mapping studies. More importantly, because of a lack of a priori knowledge about epistatic loci, exploring all possible combinations of genetic variants can result in low statistical power after correcting for multiple hypothesis tests.

As a departure from the explicit search strategy, the second category of epistatic mapping methods attempts to address the previously mentioned challenges by detecting *marginal* epistasis. Specifically, instead of directly identifying individual pairwise or higher order interactions, these approaches focus on identifying variants that have a nonzero interaction effect with any other variant in the data set. For example, the “MArginal ePIstasis Test” (MAPIT) (Crawford *et al.* 2017) assesses each variant (in turn) and identifies candidate markers that are involved in epistasis without the need to identify the exact partners with which the variants interact—thus, alleviating much of the statistical power concerns and heavy computational burdens associated with explicit search-based methods. As a framework, the marginal epistatic strategy has been implemented in both linear mixed models and machine learning and has been used for case–control studies (Crawford and Zhou 2018), pathway enrichment applications (Turchin *et al.* 2020), heritability estimation (Darnell *et al.* 2022), and even extended to explore different sources of nonadditive genetic variation (e.g. gene-by-environment interactions) (Moore *et al.* 2019; Kerin and Marchini 2020b). However, despite its wide adoption, this approach can still be underpowered for traits with low heritability or “polygenic” traits which are generated by many mutations of small effect (Crawford *et al.* 2017).

To date, both the explicit search and marginal epistasis detection methodologies have only focused on analyzing one phenotype at a time. However, many previous genetic association studies have extensively shown that jointly modeling multiple phenotypes can often dramatically increase power for variant detection (Zhou and Stephens 2014). In this work, we present the “multivariate MArginal ePIstasis Test” (mvMAPIT)—a multioutcome generalization of the MAPIT model which aims to take advantage of the relationship between traits to improve the identification of variants involved in epistasis. We formulate mvMAPIT as a multivariate linear mixed model (mvLMM) and extend a previously developed variance component estimation algorithm for efficient parameter inference and *P*-value computation in the multitrait setting (Zhou 2017). Together with reasonable model approximations, our proposed approach is scalable to moderately sized GWA studies. With detailed simulations, we illustrate the benefits of mvMAPIT in terms of providing effective type I error control and compare its power to the univariate (or single-trait) mapping strategy used in the original MAPIT model. Here, part of our main contribution is the demonstration that the power in our proposed multivariate approach is driven by the correlations between the effects of pairwise interactions on multiple traits. To close, we also apply the mvMAPIT framework to protein sequence data from a nearly combinatorially complete library of two broadly neutralizing anti-influenza antibodies (Phillips *et al.* 2021) and to 15 quantitative hematology traits assayed in a heterogeneous stock of mice from Wellcome Trust Centre for Human Genetics (Valdar, Flint, *et al.* 2006; Valdar, Solberg, *et al.* 2006).

## Materials and methods

### The marginal epistasis test for single traits

The original motivation behind the original “MArginal ePIstasis Test” (MAPIT) was to identify variants that are involved in epistasis while avoiding the need to explicitly conduct an exhaustive search over all possible pairwise interactions (Crawford *et al.* 2017). In this section, we give a statistical overview of the univariate version of MAPIT where the objective is to search for marginal epistatic effects (i.e. the combined pairwise interaction effects between a given variant and all other variants) that drive the genetic architecture of single traits. To begin, consider a GWA study with *N* individuals who have been genotyped for *J* single nucleotide polymorphisms (SNPs) encoded as {0,1,2} copies of a reference allele at each locus. In the MAPIT framework, we examine one SNP at a time (indexed by *j*) and consider the following linear model:


(1) $$y=\mu +x_{j}\beta_{j}+\sum_{l\neq j}x_{l}\beta_{l}+\sum_{l\neq j}(x_{j}\circ x_{l})\alpha_{l}+\varepsilon ,\;\varepsilon ∼N(0,\tau^{2}I),$$

where y is an *N*-dimensional vector of phenotypic states for a quantitative trait of interest measured in the *N* individuals; μ is an intercept term; X denotes an N×J matrix of genotypes with $x_{j}$ and $x_{l}$ representing *N*-dimensional vectors for the *j*th and *l*th SNPs; $\beta_{j}$ and $\beta_{l}$ are the respective additive effects; $x_{j}\circ x_{l}$ denotes the Hadamard (elementwise) product between two genotypic vectors with corresponding interaction effect size $\alpha_{l}$; ε is a normally distributed error term with mean zero and scale variance term $\tau^{2}$; and I denotes an N×N identity matrix. For convenience, we will assume that both the genotype matrix (columnwise) and trait of interest have been mean-centered and standardized. It is also worth noting that, while we limit the above to the task of identifying second-order (i.e. pairwise) interactions between genetic variants, extensions of MAPIT to higher order epistatic and gene-by-environmental effects have been shown to be straightforward to implement (Moore *et al.* 2019; Kerin and Marchini 2020a, 2020b; Zhu *et al.* 2022).

#### Variance component model formulation

Since many modern GWA applications present scenarios that would make equation (1) an underdetermined linear system (e.g. in modern biobank scale studies where genotyped markers J>N individuals), the MAPIT framework follows other standard approaches (Yang *et al.* 2010; Wu *et al.* 2011; Zhou *et al.* 2013; Bulik-Sullivan *et al.* 2015; Crawford *et al.* 2017) to ensure model identifiability by assuming that the additive and interaction effect sizes follow univariate normal distributions where $\beta_{l}∼N(0,\omega^{2}/(J-1))$ and $\alpha_{l}∼N(0,\sigma^{2}/(J-1))$ for l≠j, respectively. This key normal assumption on the regression coefficients allows for equation (1) to be equivalently represented as the following variance component model:


(2) $$y=\mu +x_{j}\beta_{j}+m_{j}+z_{j}+\varepsilon ,\;\varepsilon ∼N(0,\tau^{2}I),$$

where, in addition to previous notation, $m_{j}=\sum_{l\neq j}x_{l}\beta_{l}$ is the combined additive effects from all variants other than the *j*th and $z_{j}=\sum_{l\neq j}(x_{j}\circ x_{l})\alpha_{l}$ denote the summation of all pairwise interaction effects between the *j*th variant and all other variants. Under the variance component formulation in equation (2), the two random effects can also be expressed probabilistically as $m_{j}∼N(0,\omega^{2}K_{j})$ where $K_{j}=X_{-j}X_{-j}^{⊺}/(J-1)$ is an additive genetic relatedness matrix that is computed using all genotypes other than the *j*th SNP, and $z_{j}∼N(0,\sigma^{2}G_{j})$ where $G_{j}=D_{j}K_{j}D_{j}$ is a nonadditive relatedness matrix computed based on all pairwise interaction terms involving the *j*th SNP. Here, we let $D_{j}=\mathrm{diag}(x_{j})$ denote an N×N diagonal matrix with the *j*th genotype as its only nonzero elements. It is also important to note that both $K_{j}$ and $G_{j}$ change with every new *j*th marker that is tested.

#### Univariate point estimates

The key takeaway from the variance component model formulation in equation (2) is that $\sigma^{2}$ represents a measure on the marginal epistatic effect for each variant in the data. Therefore, in order to identify variants that have significant nonzero interaction effects, we must assess the null hypothesis $H_{0}\;:\;\sigma^{2}=0$ for each variant in the data set. The original MAPIT framework uses a computationally efficient method of moments algorithm called MQS (Zhou 2017) to estimate model parameters and to carry out calibrated statistical tests. Briefly, MQS produces point estimates that are mathematically identical to the Haseman-Elston (HE) cross-product regression (Lee *et al.* 2011; Zhou 2017; Zhu and Zhou 2020). To implement this algorithm, we first specify a two-dimensional matrix $b_{j}=[1,x_{j}]$ with 1 being an *N*-dimensional vector of ones. Next, we then multiply both sides of equation (2) by a variant-specific projection $P_{j}=I-b_{j}(b_{j}^{⊺}b_{j}{)}^{-1}b_{j}^{⊺}$ which maps the model onto a column space that is orthogonal to both the intercept and the genotypic vector $x_{j}$. This process simplifies the model specification of MAPIT in equation (2) to the following


(3) $$y_{j}^{*}=m_{j}^{*}+z_{j}^{*}+\varepsilon_{j}^{*},\;m_{j}^{*}∼N(0,\omega^{2}K_{j}^{*}),\;z_{j}^{*}∼N(0,\sigma^{2},G_{j}^{*}),\;\varepsilon_{j}^{*}∼N(0,\tau^{2}P_{j}),$$

where we denote $y_{j}^{*}=P_{j}y$; $m_{j}^{*}=P_{j}m_{j}$; $K_{j}^{*}=P_{j}K_{j}P_{j}$; $z_{j}^{*}=P_{j}^{*}z_{j}$; $G_{j}^{*}=P_{j}G_{j}P_{j}$; and $\varepsilon_{j}^{*}=P_{j}\varepsilon$, respectively. The estimators for the variance components in equation (3) are naturally based on the second moment matching equations where, in expectation, we have


(4) $$E[y_{j}^{*⊺}Hy_{j}]=\sum_{k=1}^{3}\mathrm{tr}(H\Sigma_{\;jk})\delta_{k}$$

with H being a symmetric and nonnegative definite matrix used to create weighted second moments, tr(∙) denotes the trace of a matrix, and we use shorthand to represent $\left[\Sigma_{\;j1};\Sigma_{\;j2};\Sigma_{\;j3}]=[K_{j}^{*};G_{j}^{*};P_{j}\right]$ and $\delta =(\omega^{2},\sigma^{2},\tau^{2})$, respectively. In practice, we replace the left hand side of equation (4) with the realized value $y_{j}^{*⊺}Hy_{j}$. Note that many choices of H will yield unbiased estimates for $\left(\omega^{2},\sigma^{2},\tau^{2}\right)$, but different choices of H can affect statistical efficiency of the estimates. The set of moment matching equations in MQS is generated by using the covariance matrices corresponding to the variance components in place of the arbitrary H. This system of equations can then be rewritten as the following matrix multiplication


(5) $$\delta =S^{-1}q,\;q_{k}=y_{j}^{*⊺}\Sigma_{\;jk}y_{j},\;S_{rs}=\mathrm{tr}(\Sigma_{\;jr}\Sigma_{\;js}),$$

where q is a three-dimensional vector and S is a 3×3-dimensional matrix with k,r,s∈{1,2,3} being indices to represent the different variance components. If we subset just to compute an estimate for the marginal epistatic variance component (i.e. for the second index), then equation (5) reduces to the following formula


(6) $${\hat{\sigma }}_{j}^{2}=y_{j}^{*⊺}H_{j}y_{j}^{*},$$

where the variant-specific matrix $H_{j}=(S^{-1}{)}_{21}K_{j}^{*}+(S^{-1}{)}_{22}G_{j}^{*}+(S^{-1}{)}_{23}P_{j}$ is now used in place of the arbitrary H.

#### Univariate hypothesis testing

In general, there are two ways to compute *P*-values in the MAPIT framework (Crawford *et al.* 2017). The first option uses a two-sided z-score or normal test. This particular test only requires the variance component estimate ${\hat{\sigma }}_{j}^{2}$ from equation (6) and its corresponding standard error, which is approximated in the MQS approach as


(7) $$V[{\hat{\sigma }}_{j}^{2}]\approx 2y_{j}^{*⊺}H_{j}^{⊺}V_{j}H_{j}y_{j}^{*},$$

where $V_{j}={\hat{\omega }}_{j}^{2}K_{j}^{*}+{\hat{\sigma }}_{j}^{2}G_{j}^{*}+{\hat{\tau }}_{j}^{2}P_{j}$. The second option for deriving *P*-values in the MAPIT framework uses an exact test which is based on the fact that the MQS variance component estimate follows a mixture of chi-square distributions under the null hypothesis. This is derived from both the normality assumption on $y^{*}$ and the quadratic form of the statistic in equation (6). Namely, ${\hat{\sigma }}_{j}^{2}∼\sum_{i=1}^{N}\lambda_{i}\chi_{1,i}^{2}$ where $\chi_{1}^{2}$ are chi-square random variables with one degree of freedom and $\left(\lambda_{1},\ldots ,\lambda_{N}\right)$ are the eigenvalues of the matrix


(8) $$({\hat{\omega }}_{0}^{2}K_{j}^{*}+{\hat{\tau }}_{0}^{2}P_{j}{)}^{1/2}H_{j}({\hat{\omega }}_{0}^{2}K_{j}^{*}+{\hat{\tau }}_{0}^{2}P_{j}{)}^{1/2}$$

with $\left({\hat{\omega }}_{0}^{2},{\hat{\tau }}_{0}^{2}\right)$ being the MQS estimates of $\left({\hat{\omega }}^{2},{\hat{\tau }}^{2}\right)$ under the null hypothesis $H_{0}\;:\;\sigma^{2}=0$. Several approaches have been proposed to obtain *P*-values under a mixture of chi-square distributions, including the Davies method (Davies 1980) (see Data and Software Availability). In practice, while the Davies method is an exact test and is expected to produce calibrated *P*-values, it can become computationally intensive since it scales cubically in the number of individuals *N*. On the other hand, while the normal test only scales quadratically in *N* because of the standard error approximation in equation (7), it has been shown to lead to miscalibrated *P*-values for data sets with small sample sizes. As a result, MAPIT uses a hybrid procedure which implements the normal test by default, and then applies the Davies method when the *P*-value from the normal test is below the nominal threshold of 0.05 (Crawford *et al.* 2017).

### Derivation of the multivariate marginal epistasis test

The “multivariate MArginal ePIstasis Test” (mvMAPIT) is a multioutcome extension of the statistical framework MAPIT which aims to identify variants that are involved in epistatic interactions by leveraging the covariance structure of nonadditive genetic variation that is shared between multiple traits. Once again, consider a GWA study with *N* individuals—however, this time, assume that each observation has been measured for *D* different phenotypes. We will denote these sets of outcomes via a D×N-dimensional matrix $Y=[y_{1}^{⊺},\ldots ,y_{D}^{⊺}]$ with $y_{d}$ denoting an *N*-dimensional phenotypic vector for the *d*th trait. Given the *j*th variant of interest, we specify the mvMAPIT approach as the following multivariate linear mixed model (mvLMM) (Zhou and Stephens 2014)


(9) $$Y=U+\beta_{j}x_{j}^{⊺}+\sum_{l\neq j}\beta_{l}x_{l}^{⊺}+\sum_{l\neq j}\alpha_{l}(x_{j}\circ x_{l}{)}^{⊺}+E\;E∼MN(0,V_{\varepsilon },I),$$

where, in addition to previous notation, U is a D×N-dimensional matrix which contains population-level intercepts that are the same for all individuals within each trait; $\beta_{j}$ and $\beta_{l}$ are *D*-dimensional vectors of additive effects for the *j*th and *l*th genotypic vectors; $\alpha_{l}$ is a *D*-dimensional vector of coefficients for the interaction effects between the *j*th and *l*th SNPs spanning all traits; and E denotes an D×N matrix of residual errors that is assumed to follow a matrix-variate normal distribution with mean 0, within column covariance $V_{\varepsilon }$ among the *D* traits, and independent within row covariance among the *N* individuals in the study.

Similar to the univariate setting, we need to make additional probabilistic assumptions to ensure model identifiability when equation (9) is an underdetermined linear system. To that end, let $B=[\beta_{l}{]}_{l\neq j}$ and $A=[\alpha_{l}{]}_{l\neq j}$ denote the collection of coefficients not involving the *j*th variant of interest. Here, we will assume that these D×(J−1) effect size matrices also follow matrix-variate normal distributions where $B∼MN(0,V_{\beta },I)$ and $A∼MN(0,V_{\alpha },I)$, respectively. Note that this formulation is largely similar to the univariate case except with the additional property that the phenotypes being studied share some genetic covariance through $V_{\beta }$ and $V_{\alpha }$. This assumption, coupled with the affine transformation property of matrix normal distributions, allows for us to equivalently represent the mvMAPIT model in equation (9) as the following multivariate variance component model:


(10) $$Y+U+\beta_{j}x_{j}^{⊺}+M_{j}+Z_{j}+E,\;E∼MN(0,V_{\varepsilon },I),$$

where $M_{j}=\sum_{l\neq j}\beta_{l}x_{l}^{⊺}$ with $M_{j}∼MN(0,V_{\beta },K_{j})$ represents the combined additive effects from all other variants across the *D* traits and $Z_{j}=\sum_{l\neq j}\alpha_{l}(x_{j}\circ x_{l}{)}^{⊺}$ with $Z_{j}∼MN(0,V_{\alpha },G_{j})$ encodes all pairwise interaction terms involving the *j*th SNP across the *D* traits. Here, the term $Z_{j}$ becomes the main focus of model inference.

As we will show below, mvMAPIT works by analyzing pairs of traits at a time; this allows the framework to be applied to any number of measured phenotypes. Analyzing more traits simply requires more computational resources both in terms of wall-clock time and computer memory. For each point estimate, mvMAPIT performs matrix operations that scale quadratically with sample size. The software also needs to store covariance matrices corresponding to the number of random effects in the model. Both these added costs scale as D(D+1)/2 for *D* traits. If one were to also consider higher order interactions, an additional resource burden would come from requiring additional covariance matrices to be stored as well as projecting these covariance matrices onto a space that is orthogonal to the variant of interest and the population intercept. In the multivariate setting, the time complexity of the projection step scales on the order of $DN^{2}$ with again *N* being the number of samples in the data.

### Hypothesis testing in the mvMAPIT framework

The goal of the mvMAPIT framework still comes down to assessing the null hypothesis that tests for nonzero marginal epistatic effects. However, parameter estimation in mvLMMs can present substantial computational challenges. For example, one common way in the literature to rewrite the model specified in equation (10) is to vectorize (or stack) the columns of each matrix in the regression such that y=vec(Y), μ=vec(U), $m_{j}=\mathrm{vec}(M_{j})$, $z_{j}=\mathrm{vec}(Z_{j})$, and ε=vec(E). Under this reformulation, we could simply follow the procedures in equations (3)–(8) to find significant variance components; but since $V[m_{j}]=K_{j}⊗V_{\beta }$ and $V[z_{j}]=G_{j}⊗V_{\alpha }$ are each ND×ND dimensions (via the Kronecker product ⊗), the periterative computation time for performing hypothesis testing on each *j*th SNP would now increase both with the number of individuals (*N*) and with the number of phenotypes (*D*). This could make model fitting infeasible for large biobanks even with only two traits. As an alternative, we present a combinatorial approach which first fits univariate MAPIT models and then combines the resulting *P*-values with those stemming from a “covariance statistic” which looks for shared marginal epistatic effects between all pairwise combinations of the *D* traits. Importantly, our combinatorial approach does not make assumptions about the covariance structure between traits, which would need to be known (or assumed) in the Kronecker formulation.

To implement the multivariate marginal epistasis test, we follow a similar strategy used in the univariate MAPIT model and right multiply equation (10) by a variant-specific projection $P_{j}=I-b_{j}(b_{j}^{⊺}b_{j}{)}^{-1}b_{j}^{⊺}$ which maps the model onto a column space that is orthogonal to the population-level intercepts and the genotypic vector $x_{j}$. This results in a simplified mvLMM of the following form:


(11) $$Y_{j}^{*}=M_{j}^{*}+Z_{j}^{*}+E_{j}^{*},\;E_{j}^{*}∼MN(0,V_{\varepsilon },P_{j}),$$

where, in addition to previous notation, $Y_{j}^{*}=YP_{j}$; $M_{j}^{*}=M_{j}P_{j}$; $Z_{j}^{*}=Z_{j}P_{j}$; and $E_{j}^{*}=E_{j}P_{j}$, respectively. Probabilistically, this transformation assumes $M_{j}^{*}∼MN(0,V_{\beta },K_{j}^{*})$ with $K_{j}^{*}=P_{j}K_{j}P_{j}$; and $Z_{j}^{*}∼MN(0,V_{\alpha },G_{j}^{*})$ with $G_{j}^{*}=P_{j}G_{j}P_{j}$. The joint analysis of multiple outcomes requires a generalization of the MQS algorithm to also include moment estimates for the covariance components between traits. Let $y_{c}^{*}$ and $y_{d}^{*}$ be the *c*th and *d*th rows of the adjusted phenotypic matrix $Y_{j}^{*}$, respectively. The general MQS estimates for the marginal epistatic effect is a generalization of equation (6) which is given in the following quadratic form:


(12) $${\hat{\sigma }}_{\;j,(cd)}^{2}=y_{c}^{*⊺}H_{j}y_{d}^{*},$$

where $H_{j}$ is as previously defined in the univariate MAPIT case and the trait-specific indices span between the c,d∈1,…,D phenotypes. Here, when c=d, the above is exactly equal to equation (6) and the variance component point estimate is computed using only one trait row in $Y_{j}^{*}$. On the other hand, when c≠d, equation (12) takes on a bilinear form where $E[y_{c}^{*⊺}H_{j}y_{d}^{*}]=\mathrm{tr}(H_{j}V_{\;j,(cd)})$ with $V_{\;j,(cd)}=V[y_{c}^{*},y_{d}^{*}]$ being the covariance between any two traits of interest. The corresponding standard error of the bilinear covariance component can then be estimated via the following approximation (Searle 1979):


(13) $$V[{\hat{\sigma }}_{\;j,(cd)}^{2}]\approx y_{c}^{*⊺}H_{j}^{⊺}V_{\;j,(cd)}H_{j}y_{d}^{*}+y_{c}^{*⊺}H_{j}^{⊺}V_{\;j,(dd)}H_{j}y_{c}^{*}.$$

Once again, notice that when c=d, the term $V_{\;j,(cd)}=V_{\;j,(dd)}$ and the above approximation in equation (13) is equal to equation (7).

The combinatorial hypothesis testing procedure that is used in mvMAPIT occurs in three key steps:

1. In the first step, the model fits univariate models for all *D* traits of interests (i.e. using equations (3)–(8) from the MAPIT model or equivalently equations (12) and (13) with c=d). Here, we use the proposed hybrid testing approach where we first implement a normal test by default, and then apply the exact Davies method when the *P*-value from the normal test is below the nominal significance threshold of 0.05 (Crawford *et al.* 2017).
2. In the second step, we derive *P*-values for the covariance components (i.e. using equations (12) and (13) when c≠d) with a normal test. As we will show below, the *P*-values derived for the covariance components with the asymptotic normal approximation tend to be slightly deflated under the null hypothesis. While this leads to generally conservative behavior with respect to type I error control, the downside is that the test may result in reduced power under the alternative, especially after multiple correction for data sets with small sample sizes or for traits that have low genetic correlation. In these cases, deriving an exact test to obtain more calibrated *P*-values could be done; however, we do not explore this line of work here.
3. In the third and final step, mvMAPIT combines the *P*-values from the first two steps into an overall marginal epistatic *P*-value. Assume that we have T=3 sets of *P*-values (two sets corresponding to marginal effects and one covariance set). The mvMAPIT software carries out the *P*-value combining procedure in three different ways. The first assumes that each of the t=1,…,T tests are (effectively) independent and implements Fisher’s method (Fisher 1925) which combines *P*-values into a single chi-square test statistic using the formula $\chi_{2T}^{2}∼-2\sum_{t=1}^{T}\log(p_{t})$, where $p_{t}$ denotes the *P*-value from the *t*th test. In Fisher’s method, the $\chi^{2}$ test statistic will be large when *P*-values tend to be small (i.e. when the null hypothesis is not true for every test). The second approach assumes an unknown dependency structure between each of the *T* tests and computes a harmonic mean (Wilson 2019a) *P*-value, where $\;p=\sum_{t}w_{t}/\sum_{t}w_{t}/p_{t}$. Here, $\sum_{t}w_{t}=1$ are weights which we uniformly set to be $w_{t}=1/T$ for all *P*-values. The last approach implements the Cauchy combination test which produces analytic *P*-values for any arbitrary dependency structure (Liu and Xie 2020). Under this method, the Cauchy combination test statistic is defined as $CCT=\sum_{t}w_{t}\text{tan}\{(0.5-p_{t})\pi \}$, where tan(∙) is the tangent function and again we set $w_{t}=1/T$ to be uniform for all *P*-values. The test statistic follows a standard Cauchy distribution CCT∼C(0,1).

In practice, epistatic effects are assumed to make small contributions to the overall broad-sense heritability of complex traits (Hivert *et al.* 2021; Pazokitoroudi *et al.* 2021; Wainschtein *et al.* 2022). As a result, detecting associated variants that significantly contribute to nonadditive variation can be difficult. Intuitively, this combinatorial approach is meant to aggregate over the signal identified in both the marginal and covariance tests to improve power. In our results below, we show that Fisher’s method, the harmonic mean, and the Cauchy combination test approach are well calibrated under the null hypothesis (i.e. only additive effects for all traits analyzed) and increase the ability to detect marginal epistatic variants under the alternative in both simulations and real data.

#### Note on settings where mvMAPIT is designed to be most powered

**Fig. 1.**
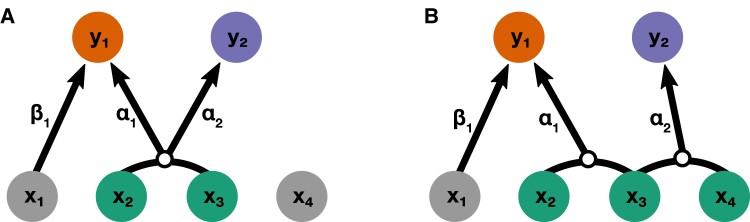
Schematic of the types of shared interactions modeled by the multivariate marginal epistasis test. Consider two simple, proof-of-concept simulation scenarios where two traits $\left(y_{1},y_{2}\right)$ are generated by a combination of four SNPs $\left(x_{1},x_{2},x_{3},x_{4}\right)$. a) First scenario where (in expectation) $E[y_{1}]=x_{1}\beta_{1}+(x_{2}\circ x_{3})\alpha_{1}$ and $E[y_{2}]=(x_{2}\circ x_{3})\alpha_{2}$. b) Second scenario where $E[y_{1}]=x_{1}\beta_{1}+(x_{2}\circ x_{3})\alpha_{1}$ and $E[y_{2}]=(x_{3}\circ x_{4})\alpha_{2}$. In both panels, variant $x_{1}$ only has an additive effect $\beta_{1}$ on trait $y_{1}$. The mvMAPIT approach models correlations between the effects of a given interaction on multiple traits. Therefore, mvMAPIT is designed to identify SNPs involved in the first scenario where the interaction between variants $x_{2}$ and $x_{3}$ is shared between traits with nonzero correlated effect sizes $\alpha_{1}$ and $\alpha_{2}$. This is in contrast to the second case, where variant $x_{3}$ is important to both traits but through distinct interactions with variants $x_{2}$ and $x_{4}$, respectively.

The formulation of the general estimates in equations (12) and (13) highlight an important takeaway in that the mvMAPIT covariance statistic models epistatic pairs that together affect the architecture of multiple traits. It is not meant to identify individual SNPs that are involved in epistasis for multiple traits while being a member of different interacting pairs. To clarify this, consider two simple scenarios in Fig. 1 where we have two phenotypes ($y_{1}$ and $y_{2}$) that are generated by a combination of four SNPs $\left(x_{1},x_{2},x_{3},x_{4}\right)$. In the first scenario, we say that (in expectation) $E[y_{1}]=x_{1}\beta_{1}+(x_{2}\circ x_{3})\alpha_{1}$ and $E[y_{2}]=(x_{2}\circ x_{3})\alpha_{2}$ (Fig. 1a), while in the second scenario, $E[y_{1}]=x_{1}\beta_{1}+(x_{2}\circ x_{3})\alpha_{1}$ and $E[y_{2}]=(x_{3}\circ x_{4})\alpha_{2}$ (Fig. 1b). The key to power in the mvMAPIT framework is that, in the first scenario, the interaction between $x_{2}$ and $x_{3}$ appears in both traits with nonzero correlation between the effect sizes $\alpha_{1}$ and $\alpha_{2}$. This is in contrast to the second scenario where there is a common variant involved in epistasis but it is a member of two different sets of interactions that affect each trait. The mvMAPIT covariance statistic captures the situation illustrated in the first scenario (Fig. 1a) but not in the second (Fig. 1b).

### Simulation study design

To test the utility of the mvMAPIT framework, we modified a frequently used simulation scheme (Crawford *et al.* 2017; Darnell *et al.* 2022) to generate collections of synthetic quantitative traits under multiple genetic architectures using real genotypes from chromosome 22 of the control samples in the Wellcome Trust Case Control Consortium (WTCCC) 1 study. After preprocessing, considering this particular group of individuals and SNPs resulted in a data set consisting of N=2,938 individuals and J=5,747 markers. In these simulations, we randomly choose 1,000 causal SNPs to directly affect D=2 phenotypes. We generate these synthetic traits via the following general multivariate linear model:


(14) $$Y=\sum_{g\in G}\beta_{g}x_{g}^{⊺}+AW^{⊺}+E,\;E∼MN(0,I,I),$$

where Y is an D×N matrix containing all the phenotypes; G represents the set of 1,000 causal SNPs; $x_{g}$ is the genotype for the *g*th causal SNP encoded as 0, 1, or 2 copies of a reference allele; $\beta_{g}$ is a *D*-dimensional vector and represent the additive effect sizes for the *g*th SNP in the *D* traits; W is an N×M matrix which holds pairwise interactions (i.e. Hadamard products) between some subset of causal SNPs; $A=[\alpha_{1},\ldots ,\alpha_{M}]$ is a D×M matrix of interaction effect sizes with $\alpha_{m}$ being *D*-dimensional epistatic coefficients for the *m*th interaction in the *d*th trait; and E is an D×N matrix of normally distributed environmental noise.

In these studies, we assume that the total phenotypic variances for both traits in Y are set to be 1. The additive and interaction effect sizes for causal SNPs are randomly drawn from matrix normal distributions where we control the correlation of effects between traits. This simplifies to us drawing coefficients as


(15) $$\beta_{g}∼N(0,V_{\beta }),\;\alpha_{m}∼N(0,V_{\alpha }),$$

where $V_{\beta }$ and $V_{\alpha }$ are D×D covariance matrices for additive effects and pairwise interactions between the phenotypes. Once these coefficients are sampled, we rescale them so that they explain a fixed proportion of the broad-sense heritability $H^{2}$. Similarly, the environmental noise matrix is rescaled such that it explains $1-H^{2}$. When generating synthetic traits, we assume that the additive effects make up ρ% of the broad-sense heritability, while the pairwise interactions make up the remaining (1−ρ)%. Alternatively, we say that the proportion of the heritability explained by additivity is $\rho H^{2}$, while the proportion of phenotypic variance explained by pairwise interactions is $(1-\rho )H^{2}$. Setting ρ=1 represents the null model where the variation of a trait is driven by solely additive effects. Here, we use the same simulation strategy used in previous studies (Crawford *et al.* 2017; Darnell *et al.* 2022) where we divide the causal variants into three groups where:

-
$G_{1}$ is a small number of SNPs with both additive and epistatic effects;
-
$G_{2}$ is a larger number of SNPs with both additive and epistatic effects;
-
$G_{3}$ is a large number of SNPs with only additive effects.

Here, the epistatic causal SNPs interact between sets. All SNPs in $G_{1}$ interact with all SNPs in the $G_{2}$, but do not interact with variants in their own group (with the same rule applies to the second group). Again, each interaction between SNPs in the two groups has an epistatic effect size $\alpha_{d}$ for the *d*th trait (see Fig. 1a). The correlation of epistatic effects between traits is determined via the correlation coefficient $v_{\alpha ,12}$ (i.e. the off-diagonal elements of $V_{\alpha }$). With this set up, one can think of the SNPs assigned to $G_{1}$ as being the “hub nodes” in an interaction network. Note that we use this setup because it has been shown that the ability to detect two interacting variants depends on the proportion of phenotypic variance that they marginally explain. For example, in our case, this means that power is expected to depend on $V[W\alpha ]/|G_{1}|$ and $V[W\alpha ]/|G_{2}|$ for groups 1 and 2, respectively, where |G| denotes the cardinality of the set. Given different parameters for the generative model in equation (14), we simulate data mirroring a wide range of genetic architectures by varying the following parameters:

- broad-sense heritability: $H^{2}=0.3$ and 0.6;
- proportion of phenotypic variation that is explained by additive effects: ρ=0.5, 0.8, and 1;
- causal SNPs in each of the three groups: $\left\{|G_{1}|,|G_{2}|,|G_{3}|\}=\{10,10,980\right\}$ and {10, 20, 970};
- correlation between additive effects: $v_{\beta ,12}=0$, 0.8, and 1;
- correlation between epistatic effects: $v_{\alpha ,12}=0$ and 0.8.

All figures and tables show the mean performances (and standard errors) for each parameter combination across 100 simulated replicates.

### Preprocessing of the heterogeneous stock of mice data set

As part of the analyses, this work makes use of GWA data from the Wellcome Trust Centre for Human Genetics (Valdar, Flint, *et al.* 2006; Valdar, Solberg, *et al.* 2006) (http://mtweb.cs.ucl.ac.uk/mus/www/mouse/index.shtml). The genotypes from this study were downloaded directly using the BGLR-R package (Perez and de los Campos 2014). This study contains N=1,814 heterogeneous stock of mice from 85 families (all descending from eight inbred progenitor strains) (Valdar, Flint, *et al.* 2006; Valdar, Solberg, *et al.* 2006), and 131 quantitative traits that are classified into 6 broad categories including behavior, diabetes, asthma, immunology, hematology, and biochemistry. Phenotypic measurements for these mice can be found freely available online to download (details can be found at http://mtweb.cs.ucl.ac.uk/mus/www/mouse/HS/index.shtml and https://github.com/lcrawlab/mvMAPIT). In the analyses below, we focused on 15 hematological phenotypes including: atypical lymphocytes (ALY; Haem.ALYabs), basophils (BAS; Haem.BASabs), hematocrit (HCT; Haem.HCT), hemoglobin (HGB; Haem.HGB), large immature cells (LIC; Haem.LICabs), lymphocytes (LYM; Haem.LYMabs), mean corpuscular hemoglobin (MCH; Haem.MCH), mean corpuscular volume (MCV; Haem.MCV), monocytes (MON; Haem.MONabs), mean platelet volume (MPV; Haem.MPV), neutrophils (NEU; Haem.NEUabs), plateletcrit (PCT; Haem.PCT), platelets (PLT; Haem.PLT), red blood cell count (RBC; Haem.RBC), red cell distribution width (RDW; Haem.RDW), and white blood cell count (WBC; Haem.WBC). All phenotypes were previously corrected for sex, age, body weight, season, year, and cage effects (Valdar, Flint, *et al.* 2006; Valdar, Solberg, *et al.* 2006). For individuals with missing genotypes, we imputed values by the mean genotype of that SNP in their corresponding family. Only polymorphic SNPs with minor allele frequency above 5% were kept for the analyses. This left a total of J=10,227 autosomal SNPs that were available for all mice.

## Results

### mvMAPIT produces calibrated *P*-values and conservative type I error rates

**Fig. 2.**
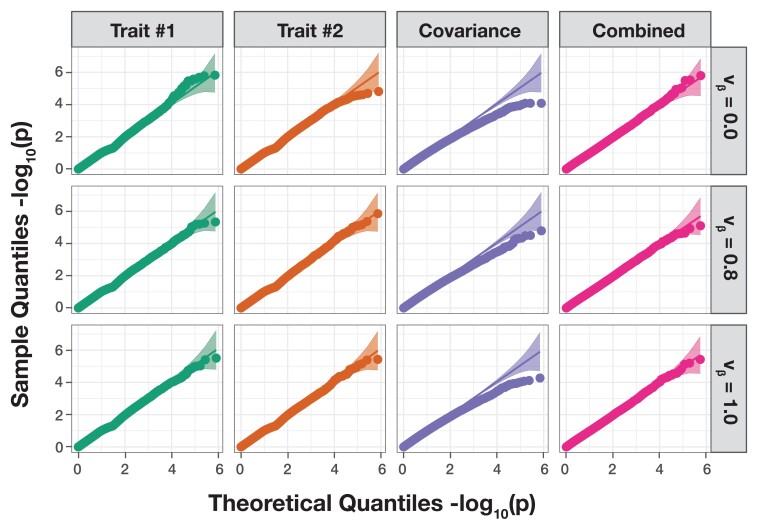
The mvMAPIT framework using Fisher’s method produces well-calibrated *P*-values when traits are generated by only additive effects (sample size N=2,500 individuals). In these simulations, quantitative traits are simulated to have narrow-sense heritability $h^{2}=0.6$ with an architecture made up of only additive genetic variation. Each row of QQ plots corresponds to a setting where the additive genetic effects for a causal SNP have different correlation structures across traits. In these simulations, we consider scenarios where we have independent traits ($v_{\beta }=0$), highly correlated traits ($v_{\beta }=0.8$), and perfectly correlated traits ($v_{\beta }=1$). The first two columns show *P*-values resulting from the univariate MAPIT test on “trait #1” and “trait #2,” respectively. The third column depicts the “covariance” *P*-values which corresponds to assessing the pairwise interactions affecting both traits. Lastly, the fourth column shows the final “combined” *P*-value which combines the *P*-values from the first three columns using Fisher’s method. The 95% confidence interval for the null hypothesis of no marginal epistatic effects is also shown as the shaded band. Each plot combines results from 100 simulated replicates.

In this section, we make use of a previously described simulation scheme (Crawford *et al.* 2017; Darnell *et al.* 2022) in order to investigate whether mvMAPIT and its combinatorial inference approach preserves the desired type I error rate and produces well-calibrated *P*-values under the null hypothesis. Here, we generate synthetic phenotypes using real genotypes from the 22nd chromosome of the control samples in the WTCCC 1 study (Burton *et al.* 2007). Altogether, these data consist of N=2,938 individuals and J=5,747 SNPs. Since the goal of mvMAPIT is to search for variants involved in epistatic interactions, we consider the null model to be satisfied when the phenotypic variation of the synthetic traits are solely driven by additive effects. Here, we first subsample the genotypes for N=1,000, 1,750, and 2,500 observations. Next, we randomly select 1,000 causal SNPs and simulate continuous phenotypes by using the linear model $Y=BX^{⊺}+E$. The additive effect sizes for each causal SNP are drawn as $\beta ∼N(0,V_{\beta })$ across traits, and then we scale all terms to ensure a narrow-sense heritability of 60%. In these simulations, we vary the correlation of the additive genetic effects such that we have traits with independent additive effects ($v_{\beta ,12}=0$), traits with highly correlated additive effects ($v_{\beta ,12}=0.8$), and traits with perfectly correlated additive effects ($v_{\beta ,12}=1$). We assess the calibration of the *P*-values that are produced by mvMAPIT during each of the three key steps in its combinatorial hypothesis testing procedure. That is, we evaluate (1) the *P*-values resulting from the univariate test on each trait, (2) the *P*-values derived from the covariance test, and (3) the final overall *P*-value that is computed by combining the first two sets of *P*-values via Fisher’s method, the harmonic mean, or the Cauchy combination procedure. Note that we expect the *P*-values from the first univariate test to be well-calibrated since it is equivalent to the MAPIT model. Figure 2 and Supplementary Figs. S1–S2 show the quantile–quantile (QQ) plots based on *P*-values combined using Fisher’s method, while Supplementary Figs. S3–S8 depict results while using the harmonic mean and the Cauchy combination test. Similarly, Table 1 and Supplementary Tables S1–S8 show the empirical type I error rates estimated for mvMAPIT at significance levels P=0.05, 0.01, and 0.001, respectively.

**Table 1.** The mvMAPIT framework using Fisher’s method preserves type I error rates under the null model when traits are generated by only additive effects (sample size N=2,500 individuals).

	Add. effect corr.	*P* = 0.05	*P* = 0.01	*P* = 0.001
Univariate	$v_{\beta }=0.0$	0.030 ($1\times 10^{-2}$)	0.009 ($2\times 10^{-3}$)	0.0010 ($9\times 10^{-4}$)
	$v_{\beta }=0.8$	0.030 ($1\times 10^{-2}$)	0.009 ($2\times 10^{-3}$)	0.0010 ($7\times 10^{-4}$)
	$v_{\beta }=1.0$	0.030 ($1\times 10^{-2}$)	0.009 ($3\times 10^{-3}$)	0.0009 ($7\times 10^{-4}$)
Covariance	$v_{\beta }=0.0$	0.040 ($1\times 10^{-2}$)	0.006 ($2\times 10^{-3}$)	0.0003 ($3\times 10^{-4}$)
	$v_{\beta }=0.8$	0.040 ($1\times 10^{-2}$)	0.007 ($2\times 10^{-3}$)	0.0004 ($5\times 10^{-4}$)
	$v_{\beta }=1.0$	0.040 ($1\times 10^{-2}$)	0.006 ($2\times 10^{-3}$)	0.0003 ($4\times 10^{-4}$)
Combined	$v_{\beta }=0.0$	0.040 ($1\times 10^{-2}$)	0.006 ($2\times 10^{-3}$)	0.0003 ($3\times 10^{-4}$)
	$v_{\beta }=0.8$	0.040 ($1\times 10^{-2}$)	0.007 ($2\times 10^{-3}$)	0.0004 ($5\times 10^{-4}$)
	$v_{\beta }=1.0$	0.040 ($1\times 10^{-2}$)	0.006 ($2\times 10^{-3}$)	0.0003 ($4\times 10^{-4}$)

In these simulations, quantitative traits are simulated to have narrow-sense heritability $h^{2}=0.6$ with an architecture made up of only additive genetic variation. Each row corresponds to a setting where the additive genetic effects for a causal SNP have different correlation structures across traits. In these simulations, we consider scenarios where we have traits with independent additive effects ($v_{\beta }=0$), traits with highly correlated additive effects ($v_{\beta }=0.8$), and traits with perfectly correlated additive effects ($v_{\beta }=1$). We assess the calibration of the *P*-values that are produced by mvMAPIT during each of the three key steps in its combinatorial hypothesis testing procedure (see *Materials and Methods*). We show type I error rates resulting from *P*-values taken from the “univariate” test on each trait independently, the “covariance” *P*-values which corresponds to assessing the pairwise interactions affecting both traits, and the final “combined” *P*-value. Results are summarized over 100 simulated replicates. Values in the parentheses are the standard deviations across replicates.

Overall, mvMAPIT conservatively controls type 1 error rate, both in the presence of nonzero correlation between additive effects on the two traits and even with small sample sizes in the data. This result holds regardless of how *P*-values are combined in the model. The QQ plots of the *P*-values for all three components in mvMAPIT follow the expected uniform distribution for the univariate and combined analysis. Notably, because of the approximations used to compute the standard error of the test statistic in equation (13), the multivariate extension of the MQS-based testing procedure in mvMAPIT can result in conservative *P*-values for the covariance components under the null.

### Improved detection of epistatic variants using mvMAPIT in simulations

We test the power of mvMAPIT across different genetic trait architectures via an extensive simulation study (see *Materials and Methods*). Once again, we generate synthetic phenotypes using real genotypes from the 22nd chromosome of the control samples in the WTCCC 1 study (Burton *et al.* 2007). As a reminder, these data consist of N=2,938 individuals and J=5,747 SNPs. In these simulations, we randomly choose 1,000 causal variants to directly affect the genetic architecture of D=2 phenotypes. All causal SNPs are assumed to have a nonzero additive effect on both traits. Next, we randomly select a set of epistatic variants from the 1,000 causal SNPs and divide them into two interacting groups (again see Materials and Methods). We will denote these groups #1 and #2 as $G_{1}$ and $G_{2}$, respectively, with |G| denoting the cardinality of the group. One may interpret the epistatic SNPs in $G_{1}$ as being the “hub nodes” in an interaction network where each of these variants interact with all of the SNPs assigned to $G_{2}$. We generate synthetic traits by using the multivariate linear model $Y=BX^{⊺}+AW^{⊺}+E$ where, in addition to previous notation, W is matrix of interactions between the SNPs assigned to the groups $G_{1}$ and $G_{2}$. The additive and interaction coefficients for causal SNP effects across traits are drawn as $\beta ∼N(0,V_{\beta })$ and $\alpha ∼N(0,V_{\alpha })$, respectively. As a final step, we scale all terms to ensure that all genetic effects explain a fixed proportion of the total phenotypic variation. We assume a wide range of simulation scenarios by varying the following parameters:

- broad-sense heritability: $H^{2}=0.3$ and 0.6;
- proportion of phenotypic variation that is explained by additive effects: ρ=0.5 and 0.8;
- number of causal SNPs assigned to the interaction groups: $\left\{|G_{1}|,|G_{2}|\}=\{10,10\right\}$ and {10, 20};
- correlation between epistatic effects: $v_{\alpha ,12}=0$ and 0.8.

All results presented in this section are based on 100 different simulated phenotypes for each parameter combination.

**Fig. 3.**
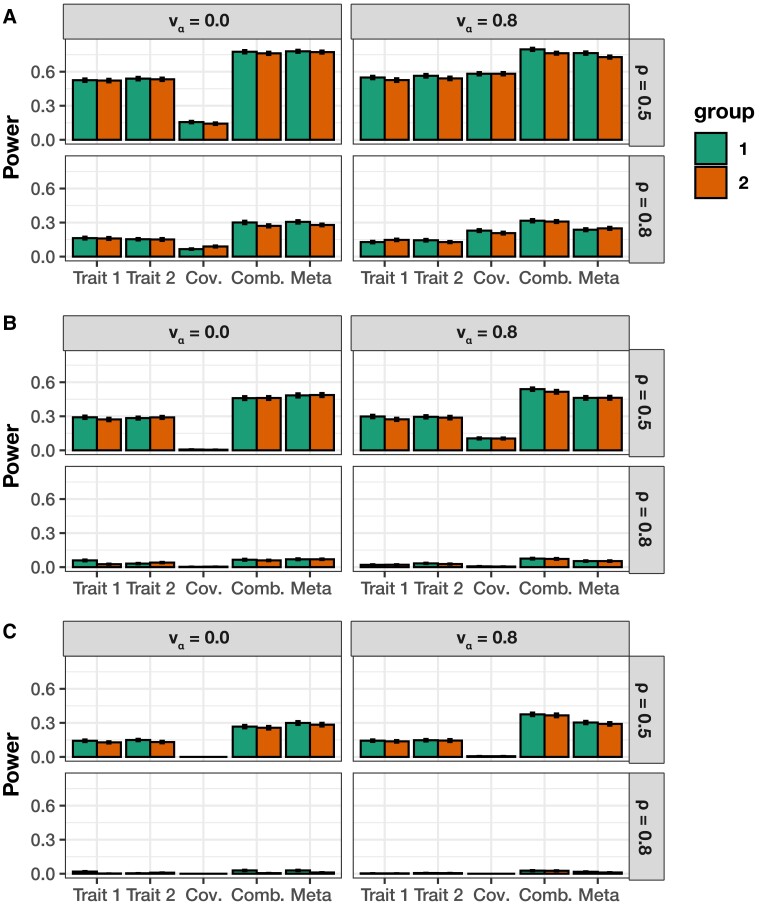
Empirical power of mvMAPIT with Fisher’s method to detect group #1 (10) and group #2 (10) epistatic variants across complex traits with moderate broad-sense heritability. In these simulations, both quantitative traits are simulated to have broad-sense heritability $H^{2}=0.6$ with architectures made up of both additive and epistatic effects. The parameter ρ={0.5,0.8} is used to determine the portion of broad-sense heritability contributed by additive effects. Each column corresponds to a setting where the epistatic effects for interactive pairs have different correlation structures across traits. In these simulations, we consider scenarios where we have traits with independent epistatic effects ($v_{\alpha }=0$) and traits with highly correlated epistatic effects ($v_{\alpha }=0.8$). This plot shows the empirical power of mvMAPIT at significance levels a) $P=5\times 10^{-2}$, b) $P=5\times 10^{-4}$, and c) $P=1\times 10^{-5}$, respectively. Results for the group #1 and #2 causal markers are shown side-by-side, respectively. For comparison, the “trait #1” and “trait #2” bars correspond to the univariate MAPIT model, the “cov” bars corresponds to power contributed by the covariance test, and “comb” details power from the overall association identified by mvMAPIT in the combination approach. Lastly, the “meta” bars shows power from the association identified by combining only the *P*-values of the two univariate cases “trait #1” and “trait #2”. Results are based on 100 simulations per parameter combination and the horizontal bars represent standard errors.

**Fig. 4.**
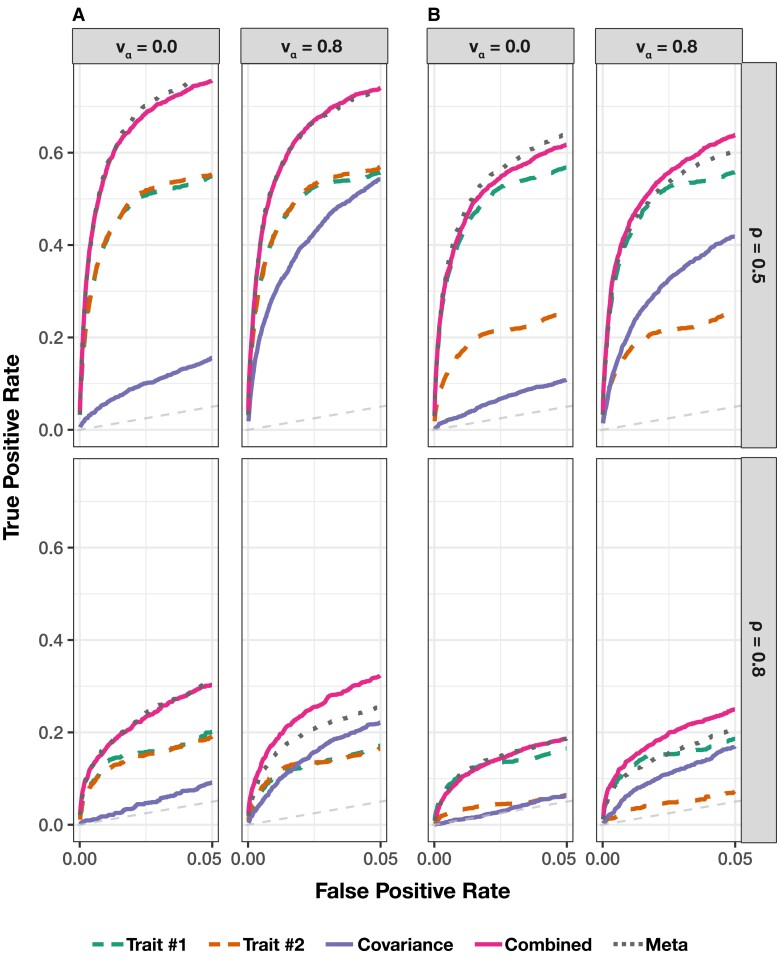
Receiver operating characteristic (ROC) curves comparing the ability of mvMAPIT with Fisher’s method to the univariate MAPIT model in detecting group #1 (10) and group #2 (10) epistatic variants across complex traits. In panel a) both traits have broad-sense heritability $H^{2}=0.6$, while in panel b) one of traits has broad-sense heritability $H^{2}=0.6$ and the other has heritability $H^{2}=0.3$. Across the rows, the parameter ρ={0.5,0.8} is used to determine the portion of broad-sense heritability contributed by additive effects. Each column corresponds to settings where the epistatic effects across traits are independent ($v_{\alpha }=0$) or highly correlated ($v_{\alpha }=0.8$). For comparison, the “trait #1” and “trait #2” dashed lines correspond to the univariate MAPIT model. The “covariance” solid line corresponds to power contributed by the covariance test. The “combined” line shows power from the overall association identified by mvMAPIT in the multivariate approach. Lastly, the “meta” dotted line shows power from the association identified by combining only the *P*-values of the two univariate cases “trait #1” and “trait #2”. Note that the upper limit of the *x*-axis (i.e. false positive rate) has been truncated at 0.05. All results are based on 100 simulated replicates.

The main point of these simulations is to highlight the potential power gained from taking a multivariate approach to epistatic detection. To that end, in each of the simulation scenarios, we assess (i) the power of running the univariate MAPIT model on each trait individually, (ii) the marginal epistatic effects detected by the covariance test, (iii) the power from the overall association identified by mvMAPIT, and (iv) a baseline meta-analytic approach which combines only the *P*-values from the univariate MAPIT models on each trait. We will hereafter refer to that last model as “meta-MAPIT” for simplicity. Figure 3 and Supplementary Figs. S9–S11 show the empirical power of the univariate MAPIT model, the covariance test, mvMAPIT, and meta-MAPIT while using Fisher’s method at various multiple hypothesis testing correction thresholds. Supplementary Figs. S12–S19 depict the same information but with mvMAPIT and meta-MAPIT using the harmonic mean and Cauchy combination test to combine *P*-values. We also compare each method’s ability to rank true positives over false positives via receiver operating characteristic (ROC) and precision-recall curves (Fig. 4 and Supplementary Figs. S20–S24). There are several key takeaways from these simulation results. Overall, the ability of the univariate MAPIT framework to detect group #1 and #2 causal variants depends on the proportion of nonadditive phenotypic variation that they explain. This has been shown in previous demonstrations of the method (Crawford *et al.* 2017). For example, when there are $|G_{2}|=10$ causal SNPs in group #2, each variant in the set is expected to explain $(1-\rho )H^{2}/10\%$ of the genetic variance. As we increase that number of causal SNPs in group #2 to $|G_{2}|=20$, this proportion of variance explained by SNPs in group #2 will decrease which will make it more difficult to prioritize markers involved in interactions. Importantly, it is worth noting that the single-phenotypic test in MAPIT depends on the total interaction effects, rather than individual pairwise effects or the number of interacting pairs. An example of this can be seen by comparing Fig. 3a to Supplementary Fig. S9a where the ability to group #1 variants is independent of the number of variants in group #2.

Intuitively, the joint modeling approach of mvMAPIT provides a viable strategy for identifying SNPs contributing to nonadditive variation that would have otherwise gone undetected by univariate methods. The scenario where mvMAPIT shows significant gains over the univariate MAPIT modeling approach is when there is nonzero correlation between the effects of the epistatic interactions shared between traits (e.g. when $v_{\alpha ,12}=0.8$). The sensitivity of the covariance hypothesis test depends on the strength of this correlation which can help increase power when combining over *P*-values in the final step of mvMAPIT. This becomes increasingly relevant in the low heritability cases. When there is no correlation of interaction effects shared between pairs of traits, combining only the univariate *P*-values in the meta-MAPIT model performs equally well as mvMAPIT which includes contributions from the covariance statistic. Figure 4 and Supplementary Figs. S20–S24 demonstrate that the sensitivity of the covariance statistic is comparable to the univariate statistic for highly correlated epistatic effects ($v_{\alpha }=0.8$) despite genetic variance being predominantly explained by additivity (ρ=0.8). In this case, the multivariate approach performs better than simply doing a meta-analysis on only the univariate *P*-values. In Fig. 4, Supplementary Figs. S10, S11, S14, S15, and S18–S24, we simulated synthetic traits such that one has a moderate broad-sense heritability $H^{2}=0.6$ and the other has heritability $H^{2}=0.3$. In these scenarios, detecting variants involved in interactions increased for the trait with low heritability. In particular, the covariance component analysis is shown to play an important role in this improved detection (e.g. see Fig. 4b).

### Synergistic epistasis in binding affinity landscapes for neutralizing antibodies

We apply the mvMAPIT framework to protein sequence data from Phillips *et al.* (2021) who generated a nearly combinatorially complete library for two broadly neutralizing anti-influenza antibodies (bnAbs), CR6261, and CR9114. This data set includes almost all combinations of one-off mutations that distinguish between germline and somatic sequences which total to J=11 heavy-chain mutations for CR6261 and J=16 heavy-chain mutations for CR9114. Theoretically, a combinatorially complete data set for 11 and 16 mutations will have 2,048 and 65,536 samples, respectively. In this particular study, we have have access to N=1,812 complete observations for CR6261 and N=65,091 complete measurements for CR9114. For our analysis with mvMAPIT, residue sequence information was encoded as a binary matrix with the germline sequence residues marked by zeros and the somatic mutations represented as ones. As quantitative traits, Phillips *et al.* (2021) measure the binding affinity of the two antibodies to different influenza strains. Here, we assess the contribution of epistatic effects when binding to $H_{1}$ and $H_{9}$ for CR6261, and $H_{1}$ and $H_{3}$ for CR9114.

**Fig. 5.**
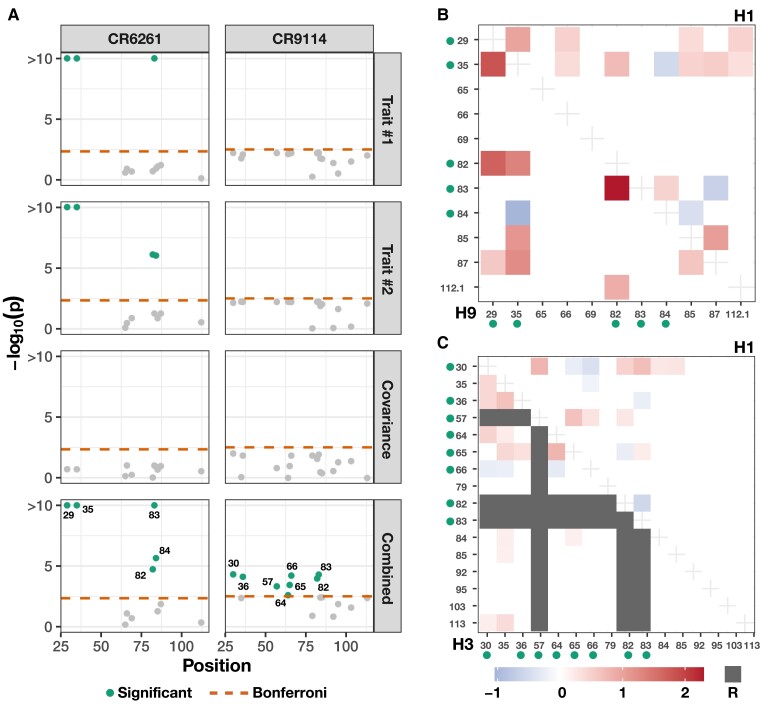
Applying mvMAPIT with Fisher’s method to broadly neutralizing antibodies recovers heavy-chain mutations known to be involved in epistatic interactions that affect binding against two influenza strains. These results are based on protein sequence data from Phillips *et al.* (2021) who generated a nearly combinatorially complete library for two broadly neutralizing anti-influenza antibodies (bnAbs), CR6261 and CR9114. For each antibody, we assess binding affinity to different influenza strains. For CR6261, traits #1 and #2 are binding measurements to the antigens $H_{1}$ and $H_{9}$, while for CR9114, we assess the same measurement for $H_{1}$ and $H_{3}$. a) Manhattan plots for the different sets of *P*-values computed during the mvMAPIT analysis. The dashed horizontal lines indicate a chain-wide Bonferroni corrected significance threshold ($P=4.55\times 10^{-3}$ for CR6261 and $P=3.13\times 10^{-3}$ for CR9114, respectively). The dots above the dashed horizontal lines are positions that have significant marginal epistatic effects after multiple correction. b, c) Exhaustive search results originally reported by Phillips *et al.* (2021). The dots next to the mutation labels on the axes are the residues that are significant in the multivariate MAPIT analysis and correspond to panel a). The shaded regions in panel b) are the regression coefficients for pairwise interactions between positions when assessing binding of CR6261with $H_{1}$ (upper right triangle) and $H_{9}$ (lower left triangle). Similarly, panel c) shows the same information when assessing binding of CR9114 with $H_{1}$ (upper right triangle) and $H_{3}$ (lower left triangle). Required mutations (indicated by R) are left out of the analysis (Phillips *et al.* 2021).

Once again, we report results after running mvMAPIT with Fisher’s method, the harmonic mean, and the Cauchy combination approach (Supplementary Table S9). Figure 5a, Supplementary Figs. S25a and S26a show Manhattan plots for *P*-values corresponding to the trait-specific marginal epistatic tests (i.e. the univariate MAPIT model), the covariance test, and the mvMAPIT approach. Here, green colored dots are positions that have significant marginal epistatic effects beyond a Bonferroni corrected threshold for multiple testing ($P=0.05/11=4.55\times 10^{-3}$ for CR6261 and $P=0.05/16=3.13\times 10^{-3}$ for CR9114, respectively). Interestingly, while the univariate MAPIT approach was able to identify significant marginal epistatic effects for CR6261, it lacked the power to identify significant positions driving nonadditive variation in binding affinity for CR9114. Overall, the combined trait approach in mvMAPIT revealed marginal epistatic effects for positions 29, 35, 82, 83, and 84 in CR6261, and positions 30, 36, 57, 64, 65, 66, 82, and 83 for CR9114. Most notably, these same positions were also identified as contributing to pairwise epistasis by Phillips *et al.* (2021). In the original study, the authors first ran an exhaustive search to statistically detect significant interactions and then conducted downstream analyses to find that these positions are likely responsible for the antibodies binding to the influenza surface protein hemagglutinin. The regression coefficients from the exhaustive search, as reported by Phillips *et al.* (2021), are illustrated in panels b and c of Fig. 5, Supplementary Figs. S25 and S26. Panel b illustrates interaction coefficients when assessing binding of CR6261with $H_{1}$ (upper right triangle) and $H_{9}$ (lower left triangle). Panel c shows the same information when assessing binding of CR9114 with $H_{1}$ (upper right triangle) and $H_{9}$ (lower left triangle). Our results show that mvMAPIT identifies all required mutations in these systems as well as most positions involved in at least one epistatic pair.

### Joint modeling of hematology traits yields epistatic signal in stock of mice

In this section, we apply mvMAPIT to individual-level genotypes and 15 hematology traits in a heterogeneous stock of mice data set from the Wellcome Trust Centre for Human Genetics (Valdar, Flint, *et al.* 2006; Valdar, Solberg, *et al.* 2006). This collection of data contains approximately N=2,000 individuals depending on the phenotype (see *Materials and Methods*), and each mouse has been genotyped at J=10,346 SNPs. As noted by previous studies, these data represent a realistic mixture of the simulation scenarios we detailed in the previous sections (i.e. varying different values of the parameter ρ). Specifically, this stock of mice is known to be genetically related with population structure and the genetic architectures of these particular traits have been shown to have different levels of broad-sense heritability with varying contributions from nonadditive genetic effects.

**Fig. 6.**
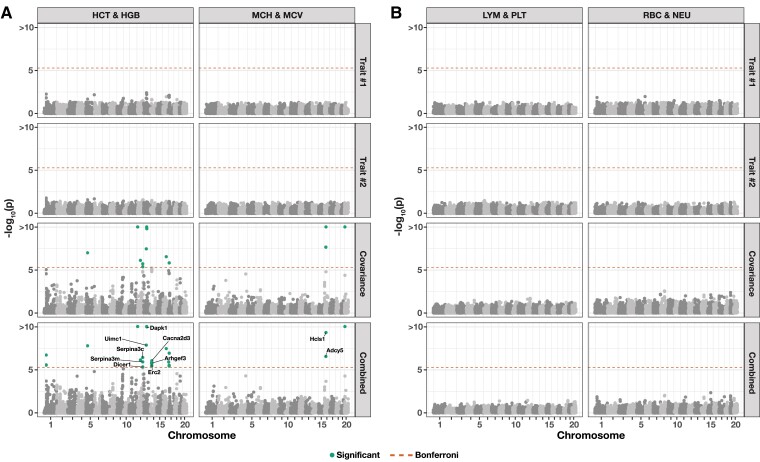
Manhattan plots of a genome-wide interaction study for two pairs of hematology traits in the heterogeneous stock of mice data set from the Wellcome Trust Centre for Human Genetics (Valdar, Flint, *et al.* 2006; Valdar, Solberg, *et al.* 2006) using mvMAPIT with Fisher’s method. Panel a) depicts results for trait pairs hematocrit (HCT) and hemoglobin (HGB) in the left column and mean corpuscular hemoglobin (MCH) and mean corpuscular volume (MCV) in the right column. Panel b) depicts results for trait pairs lymphocytes (LYM) and platelets (PLT) in the left column and red blood cell count (RBC) and neutrophils (NEU) in the right column. Here, we depict the *P*-values computed during each step of the mvMAPIT modeling pipeline. The dashed horizontal lines indicate a genome-wide Bonferroni corrected significance threshold ($P=4.83\times 10^{-6}$). The dots above the dashed horizontal lines are SNPs that have significant marginal epistatic effects after multiple test correction. Significant SNPs were mapped to the closest neighboring genes using the Mouse Genome Informatics database (http://www.informatics.jax.org) (Blake *et al.* 2020; Smith and Eppig 2009).

For each pairwise trait analysis, we provide a summary table which lists the combined *P*-values after running mvMAPIT with Fisher’s method and the harmonic mean (Supplementary Table S10). We also include results corresponding to the univariate MAPIT model and the covariance test for comparison. Overall, the single-trait marginal epistatic test only identifies significant variants for the large immature cells (LIC) after Bonferroni correction ($P=4.83\times 10^{-6}$). A complete picture of this can be seen in Supplementary Figs. S27–S29 which depict Manhattan plots of our genome-wide interaction study for all combinations of trait pairs. Here, we can see that most of the signal in the combined *P*-values from mvMAPIT likely stems from the covariance component portion of the model. This hypothesis holds true for the joint pairwise analysis of (i) hematocrit (HCT) and hemoglobin (HGB) and (ii) mean corpuscular hemoglobin (MCH) and mean corpuscular volume (MCV) (e.g. see the third and fourth rows of Fig. 6a, Supplementary Figs. S30a and S31a). One explanation for observing more signal in the covariance components over the univariate test could be derived from the traits having low heritability but high correlation between epistatic interaction effects. Recall that our simulation studies showed that the sensitivity of the covariance statistic increased for these cases. As a direct comparison, Fig. 6b, Supplementary Figs. S30b, S31b, and S32–S34 give examples of analyses that do not identify significant marginal epistatic effects even after using the mvMAPIT approach. This further supports the claim that the signal provided by the covariance statistic is not likely due to inflation.

Notably, the nonadditive signal identified by the covariance test is not totally dependent on the empirical correlation between traits (see Supplementary Fig. S35). Instead, as previously shown in our simulation study, the power of mvMAPIT over the univariate approach occurs when there is correlation between the effects of epistatic interactions shared between two traits. Importantly, many of the candidate SNPs selected by the mvMAPIT framework have been previously discovered by past publications as having some functional nonlinear relationship with the traits of interest. For example, the multivariate analysis with traits MCH and MCV show a significant SNP rs4173870 ($P=4.89\times 10^{-10}$) in the gene hematopoietic cell-specific Lyn substrate 1 (*Hcls1*) on chromosome 16 which has been shown to play a role in differentiation of erythrocytes (Castro-Ochoa *et al.* 2019). Similarly, the joint analysis of HGB and HCT shows hits in multiple coding regions. One example here are the SNPs rs3692165 ($P=1.82\times 10^{-6}$) and rs13482117 ($P=8.94\times 10^{-7}$) in the gene calcium voltage-gated channel auxiliary subunit alpha2delta 3 (*Cacna2d3*) on chromosome 14, which has been associated with decreased circulating glucose levels (IMPC 2014), and SNP rs3724260 ($P=4.58\times 10^{-6}$) in the gene *Dicer1* on chromosome 12 which has been annotated for anemia both in humans and mice (Raaijmakers *et al.* 2010).

Table 2 lists a select subset of SNPs in coding regions of genes that have been associated with phenotypes related to the hematopoietic system, immune system, or homeostasis and metabolism. Each of these are significant (after correction for multiple hypothesis testing) in the mvMAPIT analysis of related hematology traits. Some of these phenotypes have been reported as having large broad-sense heritability, which improves the ability of mvMAPIT to detect the signal. For example, the genes *Arf2* and *Cacna2d3* are associated with phenotypes related to glucose homeostasis, which has been reported to have a large heritable component (estimated $H^{2}=0.3$ for insulin sensitivity, Norris and Rich (2012)). Similarly, the genes *App* and *Pex1* are associated with thrombosis where (an estimated) more than half of phenotypic variation has been attributed to genetic effects (estimated $H^{2}\geq 0.6$ for susceptibility to common thrombosis, Souto *et al.* 2000).

**Table 2.** Notable SNPs with marginal epistatic effects after applying the mvMAPIT framework to 15 hematology traits in the heterogeneous stock of mice data set from the Wellcome Trust Centre for Human Genetics (Valdar, Flint, *et al.* 2006; Valdar, Solberg, *et al.* 2006).

SNP	Location	Trait #1	Trait #2	Trait #1 *P*-value	Trait #2 *P*-value	Cov. *P*-value	Comb. *P*-value	Gene	Genomic annotation	Reference
rs3699393	2:5887012	MCV	PLT	0.21	0.23	$5.75\times 10^{-7}$	$4.9\times 10^{-6}$	*Upf2*	Anemia and abnormal bone marrow cell development	Weischenfeldt *et al.* (2008)
rs13478092	5:3601413	LIC	PLT	0.034	0.58	$1.67\times 10^{-10}$	$1.26\times 10^{-9}$	*Pex1*	Abnormal venous thrombosis	Berendse *et al.* (2019)
rs3694887	5:102770070	ALY	LIC	$1.26\times 10^{-4}$	0.013	$2.54\times 10^{-6}$	$1.55\times 10^{-9}$	*Aff1*	Abnormal B and T cell number and morphology	Isnard *et al.* (2000)
rs3694887	5:102770070	LIC	PLT	0.013	0.28	$5.47\times 10^{-27}$	$4.49\times 10^{-26}$	*Aff1*	Abnormal B and T cell number and morphology	Isnard *et al.* (2000)
rs13478923	6:99475169	ALY	LIC	$2.8\times 10^{-4}$	0.12	$1.79\times 10^{-6}$	$1.81\times 10^{-8}$	*Foxp1*	Abnormal B cell differentiation, physiology, count	Feng *et al.* (2010), Hu *et al.* (2006)
rs13478924	6:99571626	ALY	LIC	$3.11\times 10^{-4}$	0.12	$2.70\times 10^{-6}$	$2.86\times 10^{-8}$	*Foxp1*	Abnormal B cell differentiation, physiology, count	Feng *et al.* (2010), Hu *et al.* (2006)
rs13478985	6:115245823	MCV	WBC	0.16	0.40	$1.14\times 10^{-81}$	$1.34\times 10^{-78}$	*Atg7*	Decreased bone marrow cell count	Mortensen *et al.* (2011), Clarke *et al.* (2015)
rs3723163	11:103800737	HCT	LYM	0.072	0.30	$3.99\times 10^{-107}$	$2.66\times 10^{-104}$	*Arf2*	Decreased fasting circulating glucose level	IMPC (2014)
rs3723163	11:103800737	HGB	WBC	0.069	0.25	$1.85\times 10^{-7}$	$6.76\times 10^{-7}$	*Arf2*	Decreased fasting circulating glucose level	IMPC (2014)
rs3724260	12:100163212	HGB	HCT	0.030	0.062	$1.44\times 10^{-5}$	$4.58\times 10^{-6}$	*Dicer1*	Anemia	Raaijmakers *et al.* (2010)
rs3692165	14:27756640	HCT	HGB	0.026	0.037	$9.9\times 10^{-6}$	$1.8\times 10^{-6}$	*Cacna2d3*	Decreased circulating glucose level	IMPC (2014)
rs13482117	14:27614362	HCT	HGB	0.023	0.03	$5.9\times 10^{-6}$	$9.0\times 10^{-7}$	*Cacna2d3*	Decreased circulating glucose level	IMPC (2014)
rs13482288	14:81840412	ALY	BAS	0.036	0.65	$1.78\times 10^{-8}$	$1.1\times 10^{-7}$	*Tdrd3*	Abnormal B cell differentiation and physiology	Yang *et al.* (2014)
rs4173870	16:35764290	MCH	MCV	0.14	0.71	$1.20\times 10^{-11}$	$4.89\times 10^{-10}$	*Hcls1*	Differentiation of erythrocytes	Castro-Ochoa *et al.* (2019)
rs4212102	16:84204704	PLT	WBC	0.17	0.35	$1.16\times 10^{-10}$	$2.44\times 10^{-9}$	*App*	Increased susceptibility to induced thrombosis	Xu *et al.* (2017), Souto *et al.* (2000)
rs4212186	16:84273330	PLT	WBC	0.17	0.36	$5.88\times 10^{-11}$	$1.31\times 10^{-9}$	*App*	Increased susceptibility to induced thrombosis	Xu *et al.* (2017), Souto *et al.* (2000)
rs3711994	19:45078018	ALY	LYM	$3.71\times 10^{-4}$	0.10	$1.04\times 10^{-12}$	$2.80\times 10^{-14}$	*Btrc*	Abnormal lymphocyte morphology	Nakayama *et al.* (2003)

In the first two columns, we list SNPs and their genetic location according to the mouse assembly NCBI build 34 (accessed from (Shifman *et al.* 2006)) in the format Chromosome:Basepair. Next, we give the results stemming from univariate analyses on traits #1 and #2, respectively, the covariance (cov) test, and the overall *P*-value derived by mvMAPIT using Fisher’s method. The last columns detail the closest neighboring genes found using the Mouse Genome Informatics database (http://www.informatics.jax.org) (Smith and Eppig 2009; Blake *et al.* 2020), a short summary of the suggested annotated function for those genes, and the reference to the source of the annotation. See Supplementary Table S7 for the complete list of SNP and SNP-set level results.

As a final analysis, Supplementary Figs. S36 and 37 illustrate the possibility of using the mvMAPIT framework to jointly analyze any number of traits. These show both Manhattan plots and QQ plots corresponding to the application of mvMAPIT to a subset or all of the 15 hematology traits measured in the heterogeneous stock of mice data set. In this analysis, all univariate variance and all pairwise covariance test statistics are combined. Supplementary Figure S36 shows an inflation of smaller *P*-values that is reduced by excluding traits measuring abnormal lymphocytes and large immature cells (i.e. those traits that show strong signal in for the univariate analyses; see diagonal panels in Supplementary Figs. S27–S29). Results where these two phenotypes are excluded can be found in Supplementary Fig. S37 for direct comparison.

## Discussion

The marginal epistatic testing strategy offers an alternative to traditional epistatic mapping methods by seeking to identify variants that exhibit nonzero interaction effects with any other variant in the data (Crawford *et al.* 2017; Crawford and Zhou 2018; Turchin *et al.* 2020). This framework has been shown to drastically reduce the number of statistical tests needed to uncover evidence of significant nonadditive variation in complex traits and, as a result, alleviates much of the empirical power concerns and heavy computational burden associated with explicit search-based methods. Still, models testing for marginal epistasis can be underpowered when applied to traits with low heritability or to “polygenic” traits where the interactions between mutations have small effect sizes (Crawford *et al.* 2017). In this work, we present the “multivariate MArginal ePIstasis Test” (mvMAPIT), a multioutcome extension of the univariate marginal epistatic framework (Fig. 1). Theoretically, we formulate mvMAPIT as a multivariate linear mixed model (mvLMM) where its ability to jointly analyze any number of traits relies on a generalized “variance–covariance” component estimation algorithm (Zhou 2017). Through extensive simulations, we show that mvMAPIT preserves type I error rates and produces well-calibrated *P*-values under the null model when traits are generated only by additive effects (Fig. 2 and Supplementary Figs. S1–S8, and Table 1 and Supplementary Tables S1–S8). In these simulation studies, we also show that mvMAPIT improves upon the identification of epistatic variants over the univariate test when there is correlation between the effects of genetic interactions shared between multiple traits (Figs. 3, 4, and Supplementary Figs. S9–S24). By analyzing two real data sets, we demonstrated the ability of mvMAPIT to recover heavy-chain mutations known to be involved in epistatic interactions that affect binding against two influenza strains (Phillips *et al.* 2021) (Fig. 5, Supplementary Figs. S25 and S26, and Table S9) as well as to identify hematology trait relevant epistatic SNPs in heterogeneous stock of mice (Valdar, Flint, *et al.* 2006; Valdar, Solberg, *et al.* 2006) that have also been detected in previous publications and functional validation studies (Fig. 6 and Supplementary Figs. S27–S37, and Table 2 and Supplementary Table S10). Lastly, we have made mvMAPIT an open-source R software package with documentation to facilitate its use by the greater scientific community.

We want to highlight an important caveat for mapping epistasis in real data: in genome-wide association studies, statistically inferred interactions can sometimes be explained by same-locus additive effects (Wood *et al.* 2014). This means that conclusions made by mvMAPIT, as well as any other computational method aimed at the detection of epistasis, could be confounded by additive effects of untyped or uncontrolled variants in the same region. Notably, it is difficult to control for the additive fixed effects of all variants in the same locus (Crawford *et al.* 2017). This caveat needs to be considered to guard against overinterpreting the results of mvMAPIT. The results that we present in this work (e.g. in the study of hematology trait architecture within the heterogeneous stock of mice) should be understood as an illustration of how multivariate regression frameworks can be leveraged as powerful hypothesis generating tools that can help towards resolving the true contribution of genetic effects to phenotypic variation in complex traits.

The current implementation of the mvMAPIT framework offers many directions for future development and applications. First, like other marginal epistatic mapping methods, mvMAPIT is unable to directly identify detailed interaction pairs despite being able to identify SNPs that are involved in epistasis. As shown through our simulations and real data analyses, being able to identify SNPs involved in epistasis allows us to come up with an initial (likely) set of variants that are worth further exploration, and thus represents an important first step towards identifying and understanding detailed epistatic associations. In previous studies (Zhang *et al.* 2010; Lewinger *et al.* 2013; Crawford *et al.* 2017; Pecanka and Jonker 2021), two-step ad hoc procedures have been suggested where, in our case, we would first run mvMAPIT and then focus on significant SNPs from the first step to further test all of the pairwise interactions among them in order to identify specific epistatic interaction pairs. While this approach has been shown to be effective in univariate (single-trait) analyses, this two-step procedure is still ad hoc in nature and could miss important epistatic associations. Exploring robust ways unify these two steps in a joint fashion would be an interesting area for future research. Second, in its current implementation, mvMAPIT can be computationally expensive for data sets with large sample sizes (e.g. hundreds of thousands of individuals in a biobank scale study). In this study, we develop a “variance–component component” extension to the MQS algorithm to estimate parameters in the mvMAPIT model. Theoretically, MQS is based on the method of moments and produces estimates that are mathematically identical to the Haseman-Elston (HE) cross-product regression (Lee *et al.* 2011; Zhou 2017; Zhu and Zhou 2020). In practice, MQS is not only computationally more efficient than HE regression but also provides a simple, analytic estimation form that allows for exact *P*-value computation—thus alleviating the need for jackknife re-sampling procedures (Golan *et al.* 2014) that both are computationally expensive and rely on assumptions of independence across individuals in the data (Churchill and Doerge 2008). Exploring different ways to reliably fit large-scale mvLMMs with multiple random effects is a consideration for future work. For example, as an alternative, recent studies have proposed randomized multicomponent versions of HE regression for heritability estimation which scale up to data sets with millions of individuals and variants, respectively (Wu and Sankararaman 2018; Kerin and Marchini 2020a; Pazokitoroudi *et al.* 2020). It would be interesting to develop a well-calibrated hypothesis test within the randomized HE regression setting so that it may be implemented within the mvMAPIT software for association mapping.

In the future, we plan to expand the mvMAPIT framework to also identify individual variants contributing other sources of nonadditive genetic variation such as gene-by-environment (G×E) or gene-by-sex (G×Sex) interactions. We can do this by manipulating the marginal epistatic covariance matrix in equation (10) to encode how loci interact with one or more environmental instruments (Moore *et al.* 2019; Kerin and Marchini 2020a, 2020b; Zhu *et al.* 2022). Lastly, we have focused here on applying mvMAPIT to simple quantitative traits. However, there are many important traits with significant nonadditive genetic components in plants, animals, and humans that cannot be easily reduced to simple scalar values. Examples include longitudinal traits such as growth curves (Campbell *et al.* 2018), metabolic traits such as the relative concentrations of different families of metabolites (Chan *et al.* 2011), and morphological traits such as shape or color (Demmings *et al.* 2019). Indeed, each of these traits can be decomposed into vectors of interrelated components, but treating these components as independent phenotypes within existing univariate epistatic mapping tools would be inefficient because of their statistical dependence. As an alternative, the mvMAPIT framework can be used to make joint inferences about epistasis across any number of correlated phenotypic components—which, in the case of longitudinal studies for example (Couto Alves *et al.* 2019; Wu *et al.* 2019; Cousminer *et al.* 2021; Ko *et al.* 2022), could be used to interrogate how nonadditive variation of trait architecture changes or evolves over time.

## Supplementary Material

jkad118_Supplementary_Data

## Data Availability

Source code, tutorials, and tutorials for implementing the “multivariate MArginal ePIstasis Test” are publicly available as an R package which is available online at https://github.com/lcrawlab/mvMAPIT. We use the CompQuadForm  R package (Duchesne and de Micheaux 2010) to compute *P*-values from the Davies method. The Davies method can sometimes yield a *P*-value equal exactly to 0 when the true *P*-value is extremely small (Duchesne and de Micheaux 2010). If this is of concern, one can compute the *P*-values for MAPIT using Kuonen’s saddlepoint method (Kuonen 1999) or Satterthwaite’s approximation equation (Satterthwaite 1946). In the current implementation of mvMAPIT, the saddlepoint approximation is performed if the Davies method returns with error. We wrote our own function to combine *P*-values using Fisher’s method which is largely inspired by functions in the metap  R package (Dewey 2022). We use the harmonicmeanp  R package (Wilson 2019a, 2019c) to combine *P*-values using the harmonic mean. We also write our own function to perform the Cauchy combination test based on work developed in Liu and Xie (2020). Full package documentation can be found at https://lcrawlab.github.io/mvMAPIT/. Data to reproduce figures for the broadly neutralizing antibodies as well as the mice study can be found at https://doi.org/10.7910/DVN/WPFIGU (Stamp 2022). Data about the binding affinity landscapes for neutralizing antibodies were downloaded directly from Phillips *et al.* (2021). Information about mice data set from the Wellcome Trust Centre for Human Genetics (Valdar, Flint, *et al.* 2006; Valdar, Solberg, *et al.* 2006) can be found at http://mtweb.cs.ucl.ac.uk/mus/www/mouse/index.shtml. The genotypes from this study were downloaded using the BGLR-R package (Perez and de los Campos 2014). Details about the mice phenotypes can be found at http://mtweb.cs.ucl.ac.uk/mus/www/mouse/HS/index.shtml and hematological traits can be downloaded from the mvMAPIT package. In the real data analyses, SNPs were mapped to the closest neighboring genes using the Mouse Genome Informatics database (http://www.informatics.jax.org) (Blake *et al.* 2020).
